# The Role of Electric Current-Associated Free Energy and Forced Convection on Grain Refinement in Pure Aluminum under Electropulsing

**DOI:** 10.3390/ma12233846

**Published:** 2019-11-22

**Authors:** Ning Li, Limin Zhang, Rong Zhang, Pengfei Yin, Hongjing Wu, Kaikai Song, Hui Xing

**Affiliations:** 1 Shaanxi Key Laboratory of Condensed Matter Structures and Properties and Key Laboratory of Space Applied Physics and Chemistry, Ministry of Education, School of Science, Northwestern Polytechnical University, 127 West Youyi Road, Xi’an 710072, China; lining142433@163.com (N.L.); xbwl01@mail.nwpu.edu.cn (R.Z.); wuhongjing@nwpu.edu.cn (H.W.); 2 College of Science, Sichuan Agricultural University, Ya’an 625014, China; yinpengfei@sicau.edu.cn; 3 School of Mechanical, Electrical & Information Engineering, Shandong University (Weihai), 180 Wenhua Xilu, Weihai 264209, China; songkaikai8297@gmail.com

**Keywords:** alternating electropulsing, pure aluminum, solidification, fine equiaxed grains

## Abstract

An experimental study with respect to the effect of an alternating electropulsing on grain refinement in pure aluminum was reported. The macrostructural observation with the mold preheated to different temperature and embedded the metal mesh indicated that the change of electric current-associated free energy related with the position of crystal nuclei (Δ*G*_em_) and forced convection dominated the generation of fine equiaxed grains (FEG). Under electropulsing with 480 A, Δ*G*_em_ induced the dissociation of crystal nuclei from the upper interface of the electrode and the melt, leading to the generation of FEG. For a larger current intensity, FEG originated from the dissociation of crystal nuclei on the side wall besides the upper interface due to Δ*G*_em_ and the forced convection. Furthermore, the model coupling the dissociation of crystal nuclei and dendrite fragmentation due to the forced convection and the dissociation of crystal nuclei due to Δ*G*_em_ was presented to explain the formation mechanism of FEG in pure aluminum under electropulsing.

## 1. Introduction

It is well known that replacing the large grains in castings by the fine equiaxed grains (FEG) can significantly enhance the mechanical properties of cast alloys. Over the past few decades, the different types of physical fields including the electric field [1,2,3,4,5,6,7], ultrasonic vibration [8,9,10,11,12] and electromagnetic field [13,14,15,16], have been successfully applied during solidification process of alloys for the improvement of the solidification structure. The application of electropulsing during solidification, as a promising technique for achieving fine grains, has attracted considerable attention. Consequently, extensive experimental and theoretical studies have been performed to explain the formation mechanism of FEG with electropulsing treatment [17,18,19]. Despite many hypotheses were proposed, no consensus has yet emerged.

To date, it is generally accepted that FEG is mainly achieved during the nucleation stage [3] and electromagnetical convection is essential for its generation in solidified alloys [20,21,22]. D. Räbiger et al. [3] proposed that the formation of FEG in solidified Al-Si alloy resulted solely from the global convection driven by Lorentz force under electric current. Y.H. Zhang et al. [21] further confirmed that forced convection could induce the redistribution of temperature and concentration field in the mushy zone and then accelerate dendrite fragmentation based on the improved experiments and numerical simulation. From casting experimental investigation of the relevance of the forced convection to the formation of FEG in pure aluminum under an alternating electropulsing, our previous work [20] pointed out that the forced convection gave rise to the columnar fragmentation and the dissociation of crystal nuclei on the side wall, resulting in the formation of FEG. Additionally, Y. H. Zhang et al. [5] indicated that columnar fragmentation was ascribed to the promotion of solute driven remelting of dendrite arms in low-purity aluminum due to the obvious forced convection. However, the forced convection cannot be used to explain all the structural observations accompanying grain refinement. For example, since the matrix liquid alloy has a higher electrical resistivity compared to the precipitated (Cu, Sn)-rich phase droplets, electropulsing leads to a reduction in the energy barrier to nucleation of the precipitated phase and then promotes the formation of a fine dispersed microstructure in solidified Cu-Bi-Sn immiscible alloys due to electric current-induced free energy minimization [23].

Electric current, passing through a conductor, induces an additional term *G*_e_ for the system free energy compared with that without electric current [24,25]. Experiments and theoretical analysis have proved that a significant effect of electric current-associated free energy change (Δ*G*_e_) on the microstructural improvement in materials, such as segregation of inclusions or solute precipitates in alloys [24,26], solid state phase transformations [25], fragmentation of lamellar structure of pearlitic [27] and separation of electrically neutral particles in liquids [28]. Here, it should be noted that there is a striking common feature: the formation of object has different electrical resistivities from that of the matrix. The different configurations of precipitates or inclusions in the matrix affect the distribution of electric current in the materials. Note that the different distribution of electric current corresponds to various system free energy [25,28]. It is still not sure whether electric current-induced free energy has an effect on grain refinement of alloys during solidification process. During casting, crystal nuclei first occur at, or close to, the wall of mold and present the different electrical resistivities from that of liquid alloys. The application of electropulsing may affect the distribution of crystal nuclei in liquid alloy to reduce the system free energy. This provides a possibility to utilize the driving force disturbance from electropulsing to crystal nuclei to refine the grains, while little attention is paid to the role of Δ*G*_e_ on crystal nucleus multiplication in grain refinement.

In this paper, the experimental investigation of the role of Δ*G*_e_ and the forced convection on the formation of FEG in pure aluminum was carried out. Since the solidification of pure metal is simple due to non-existent solute transport and knowledge about how electropulsing affects the solidification of pure aluminum has been accumulated by experimental and theoretical analysis, pure aluminum is selected to ensure that the formation mechanism of FEG is better clarified during the solidification process. For the work, the alternating electropulsing with frequency of 50 Hz and the effective value of current amplitude of less than 990 A was applied, since the prior work [20,29] indicated that effect of Joule heating and forced convection induced by Lorentz force on grain refinement could be distinguished by experiments with the mold embedded the wire mesh when electropulsing with 990 A was applied. It made it possible to reveal the role of Δ*G*_e_ on the formation of FEG based on the differences in electrical resistivities between the solid crystal nuclei and the liquid matrix. With regard to the complicated mechanism of grain refinement with electropulsing, we suggest that the dissociation of heterogeneous nuclei driven by Δ*G*_e_ and forced convection are two important factors, and it has important fundamental significance for the development of this technique in practice.

## 2. Experimental Procedure

**Figure 1:**
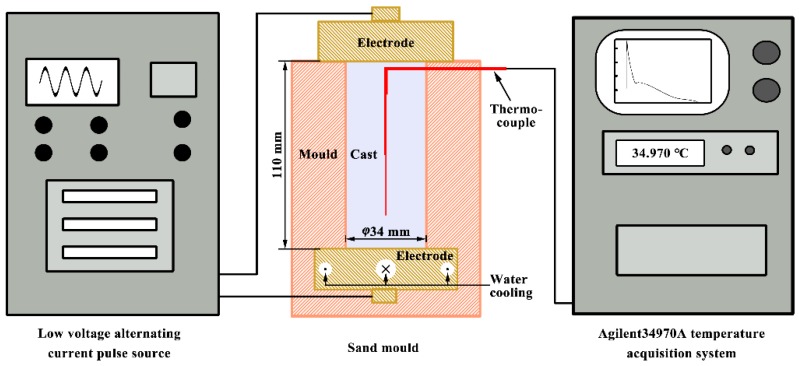
Schematic illustration of the solidification apparatus. Three thermocouples separated by 36 mm gaps were positioned along the axis of mold.

The experiments were performed in a solidification apparatus (Northwestern Polytechnical University, Xian, Shaanxi, China) incorporated with a low-voltage alternating electropulsing generator, as schematically shown in Figure 1. The bottom electrode (a rectangle copper block with 65 mm in length, 50 mm in width and 45 mm in thickness) was fixed at the bottom of cylindrical sand mold with a diameter of 34 mm and a length of 110 mm. A copper block with a size of 50 × 50 × 45 mm^3^, as the top electrode, was welded to one end of the copper rod with 8 mm in diameter. The used sand mix consists of foundry sand, clay and a little water. In the present work, the high superheating temperature of Al melt, 1173 K, is required to guarantee that the melt can pass through the stainless mesh and eliminate the heterogeneous crystal nuclei formed on the mesh. During casting experiments, pure Al (99.7%) was initially superheated to 1173 K in a graphite crucible using a resistance furnace (Shanghai Shiyan Electric Furnace Company, Shanghai, China) and kept isothermally for 30 min. Subsequently, the melt was poured into the sand mold, and then the top electrode was immediately dipped into the liquid metal with 1 mm in depth. Here, two types of experiments were performed. In the first one, the electropulsing with a frequency of 50 Hz was applied during solidification process after pouring the melt into the sand mold without preheating. For comparison, a sample without the application of electropulsing was prepared. In the second one, the sand molds were preheated to delay the formation of the solidified shell before the application of electropulsing. The effective values of applied electropulsing current were 480 and 900 A, respectively, and the corresponding current densities were 52.9 and 99.2 A·cm^−2^.

An array of K-type thermocouples was used to make temperature measurements in the mold cavity. Here, three thermocouples were placed along the ingot centerline at fixed vertical positions of 19, 55 and 91 mm above the bottom electrode. Temperature readings (at 0.2 s intervals) were recorded by a computer-aid data acquisition system. Cooling curves were immediately measured after pouring the melt. Meanwhile, the alternating electropulsing was applied during solidification.

The cylindrical ingots were sectioned along the midplane, mechanically polished using abrasive papers, and etched with an acid solution (3 mL HF, 3 mL HNO_3_, 9 mL HCl and 5 mL H_2_O) to reveal the macrostructure. Photographs of the macrostructure were taken by a Canon EOS D60 digital camera, and the grain size was measured by a mean linear intercept method.

## 3. Results and Discussion

### 3.1. Effect of Electropulsing on the Grain Macrostructure

**Figure 2:**
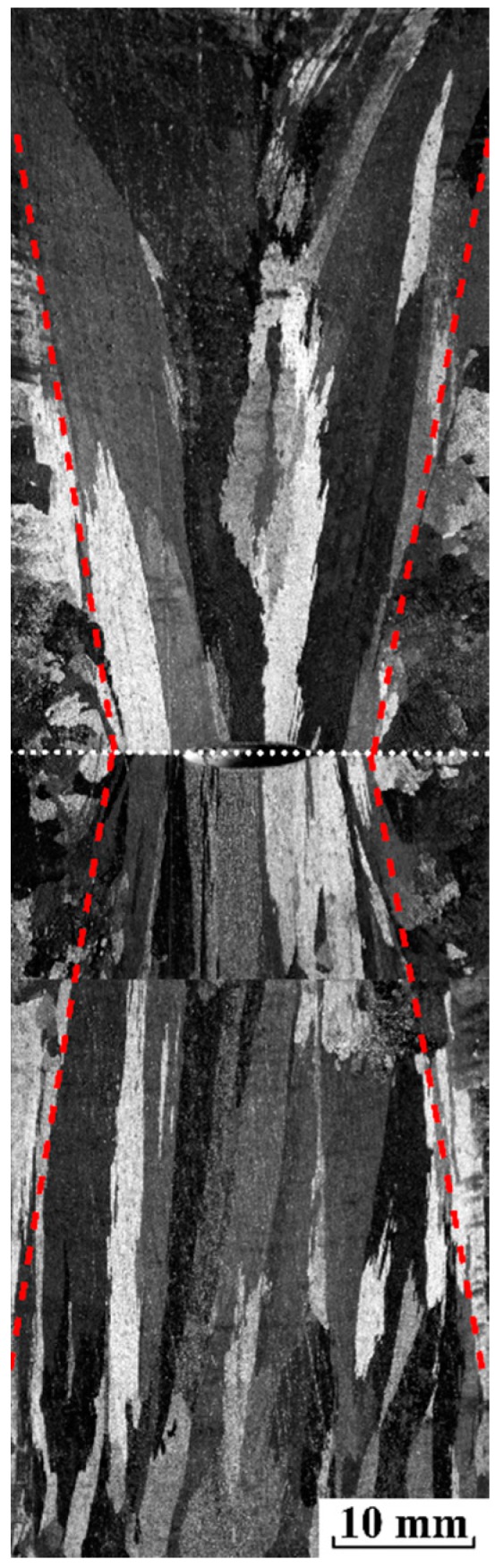
The longitudinal as-cast macrostructure of pure aluminum ingot without electropulsing.

The as-cast macrostructure of pure aluminum ingot along the longitudinal section without electropulsing is shown in Figure 2. Note that the heat flux is proportional to the metal/mold heat transfer coefficient in the casting process. Since the heat transfer coefficient between the casting surface and the copper surface at the top and bottom of the mold (~10^3^ W·m^−2^·K^−1^ [30]) at 933 K far exceeds that between the casting surface and sand mold surface (~10^2^ W·m^−2^·K^−1^ [31]), the heat is extracted mainly through the top and bottom copper electrode. As a result, the upward and downward vertical columnar grains expand over most of the ingot, and intersect at the center of ingot indicated by a dotted horizontal white line. In addition, little columnar grains grow radially towards the center of ingot, and exhibit a gradual increase in the length of the columnar grains from two ends of cylindrical mold to the axial center.

**Figure 3:**
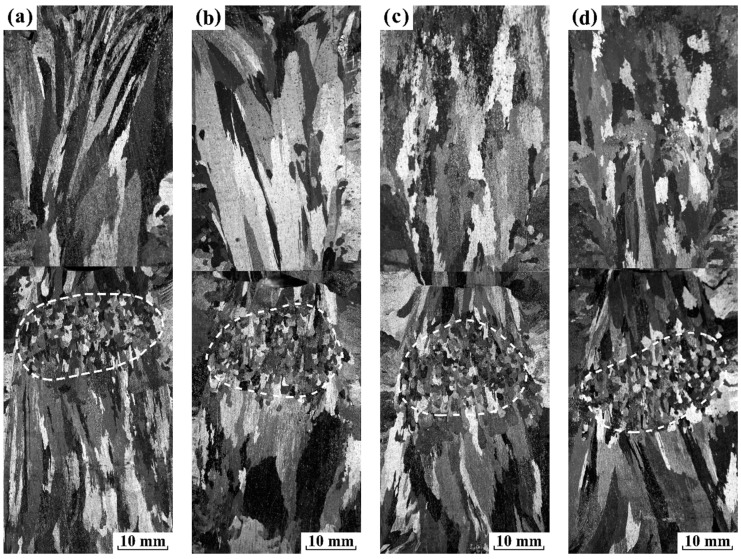
Longitudinal sections of the solidification structure in pure aluminum ingots under electropulsing with 480 A at different temperatures of mold: (**a**) room temperature; (**b**) 373 K; (**c**) 473 K and (**d**) 573 K.

**Figure 4:**
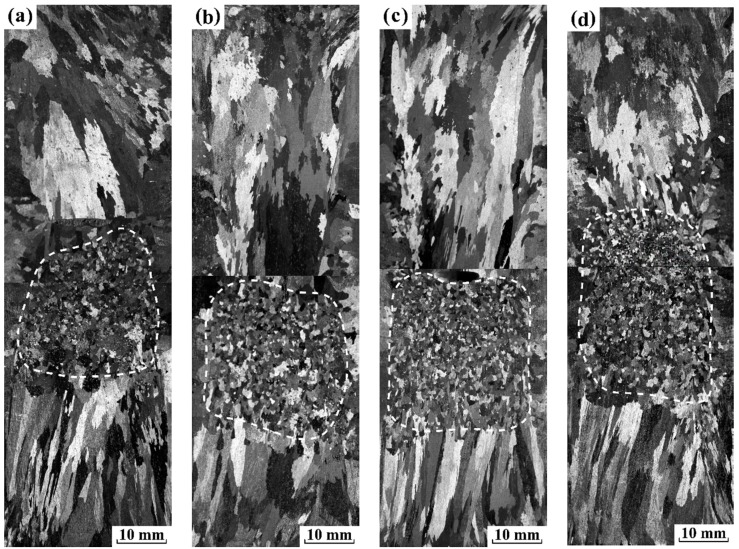
Longitudinal sections of the solidification structure in pure aluminum ingots under electropulsing with 900 A at different temperature of mold: (**a**) room temperature; (**b**) 373 K; (**c**) 473 K and (**d**) 573 K.

The sequence of macrographs in Figure 3 and Figure 4 displays, under electropulsing with 480 and 900 A, respectively, how the grain macrostructure of pure aluminum changes as a result of gradually increasing the preheating temperature of sand mold. One dotted white curve is superimposed on the macrographs to indicate the FEG zone.

**Figure 5:**
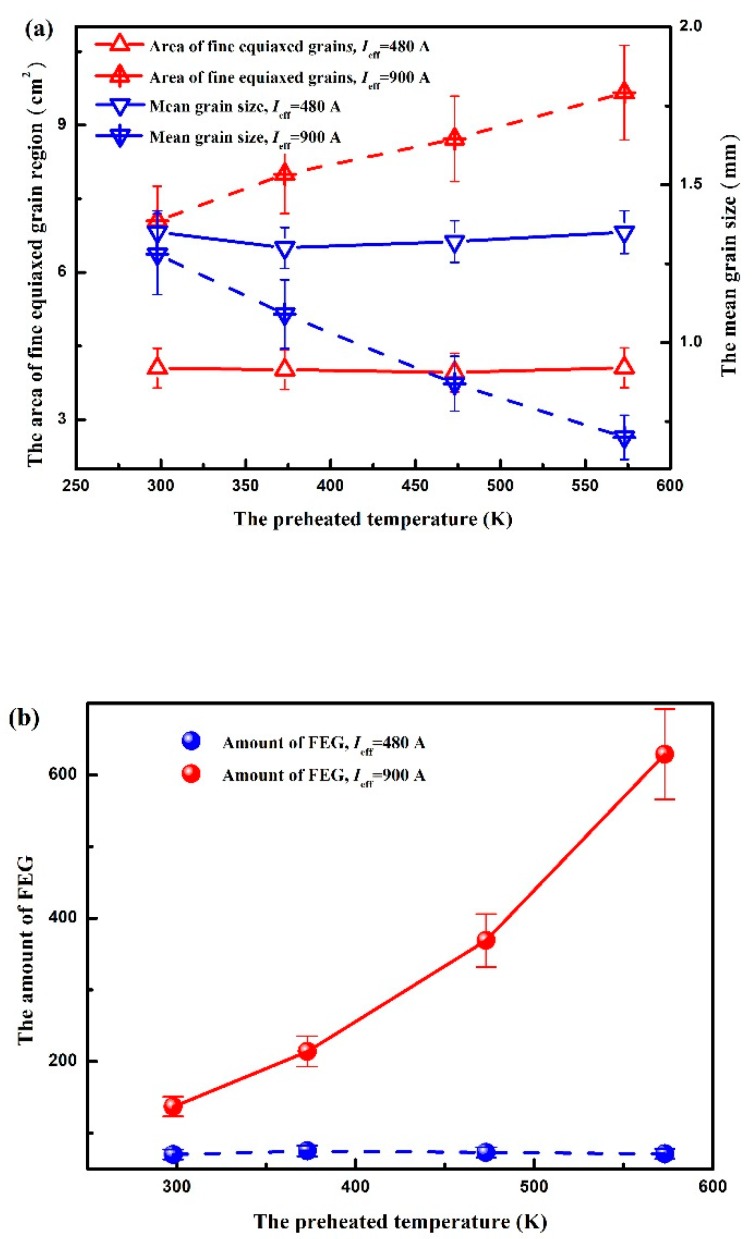
(**a**) The area of fine equiaxed grain region and the mean grain size in each of the ingots under electropulsing with 480 A and 900 A as a function of the preheating temperature of mold and (**b**) the amount of FEG in each of ingots under electropulsing with 480 A and 900 A as a function of their corresponding preheating temperature of mold.

When electropulsing with 480 A is applied to the specimen, using a mold at room temperature, the FEG region appeared in the middle and lower part of the ingot. An increase in the preheated temperature to 373 K (Figure 3b), 473 K (Figure 3c) or 573 K (Figure 3d) gives rise to a downward shift of FEG region, but the area of FEG and the mean grain size are almost unchanged, as illustrated in Figure 5. These results are different from previous observations for the application of electropulsing during the solidification of pure aluminum in which an increase in the amount of FEG as the preheated temperature of mold is increased [2,20]. In their study on an as-cast macrostructure preheating the sand mold, it indicates that more heterogeneous nuclei on the side wall dissociate and produce FEG under adequate electropulsing due to the delay of the solidified shell formation caused by preheating the mold. If we assume this to be operative, the expected macrostructure is in contradiction with the present results. It demonstrates that the dissociation of heterogeneous nuclei formed on the side wall will not happen under electropulsing with 480 A.

For a further increase in current intensity to 900 A (Figure 4) small equiaxed zone enlarges compared with that at the same preheated temperature of sand mold under electropulsing with 480 A, and the mean size of equiaxed grains becomes even more refined. In addition, the amount of FEG increases with increasing the preheated temperature of mold, as can be seen in Figure 5. As a result, the obviously different behavior to that under electropulsing of 900 A is observed for aluminum ingots under electropulsing with 480 A as the preheated temperature of mold is gradually increased. For grain refinement of pure aluminum under electropulsing, the recent studies [3,20,21] proposed that the dissociation of heterogeneous nuclei and dendrite fragmentation induced by the forced flow give rise to the multiplication of crystal nuclei. Moreover, the dissociation of heterogeneous nuclei on the side wall takes precedence over dendrite fragmentation [20]. Hence, it is speculated that the applied electropulsing with 900 A will inevitably lead to the dissociation of heterogeneous nuclei on the side wall, producing FEG. The other origin of FEG is further addressed in detail later.

### 3.2. Forced Flow Field and Relevant Thermal Analysis under Electropulsing

This subsection is focused on the forced convection caused by Lorentz force during solidification of pure aluminum under electropulsing mentioned above. Since the electromagnetical convection mainly depends on the intensity of electric current regardless of current pattern [3], the intensity of melt flow driven by electropulsing is nearly the same as that with direct current (DC) when the effective value of electropulsing, is identical to the DC amplitude. Furthermore, any effect originating from skin effect can be neglected as a result of the applied frequency small enough in our case. Under the condition of this low frequency of 50 Hz, the radial and axial components of the Lorentz force (*F*_L_ = *j* × *B*) in the melt caused by electropulsing and the induced magnetic field can be written as [32]:

(1) $$F_{\mathrm{Lr}}=\sigma (-E_{z}B_{\theta }-u_{r}B_{\theta }^{2}),$$

(2) $$F_{\mathrm{Lz}}=\sigma (E_{r}B_{\theta }-u_{z}B_{\theta }^{2}),$$

where *σ* is the electrical conductivity of the mixture of solid and liquid phase, *E*_z_ and *E*_r_ are the axial and radial components of the electric field intensity, *u*_z_ and *u*_r_ are the axial and radial components of the velocity of melt flow and *B*_θ_ is the azimuthal magnetic field. It is noted that Lorentz force is closely related to the velocity of melt flow and the electric potential. Thus, the calculation of *F*_L_ has to be coupled with the solution of momentum conservation equations. According to Equations (1) and (2), the electropulsing-driven convection in a cylindrical container is a distinct electro-vortex flow, which induces an axial jet along the direction of current density gradient for the configuration of up-down electrodes. Previous studies proposed that the electro-vortex flow plays a prominent role in the formation of FEG and macrosegregation of alloys [3,22,32,33].

To describe Lorentz force induced by an electropulsing and its driven convection, the *S* parameter that can be taken as the characteristic non-dimensional parameter is given as [34,35]:

(3) $$S=\frac{\mu_{0}I^{2}}{4\pi^{2}\rho \upsilon^{2}},$$

where *I* is the current intensity, which is the product of the current density *j* and cross-section area *s*, *ν* is the kinematic viscosity, *ρ* is the density of the liquid metal and *μ*_0_ is the vacuum permeability. Although the value of *S* can characterize the relative intensity of the flow, it is not feasible to analyze the melt flow regime utilizing the value of *S* as a criterion. As a consequence, we could not conclude the flow was laminar or turbulent in the present study.

**Figure 6:**
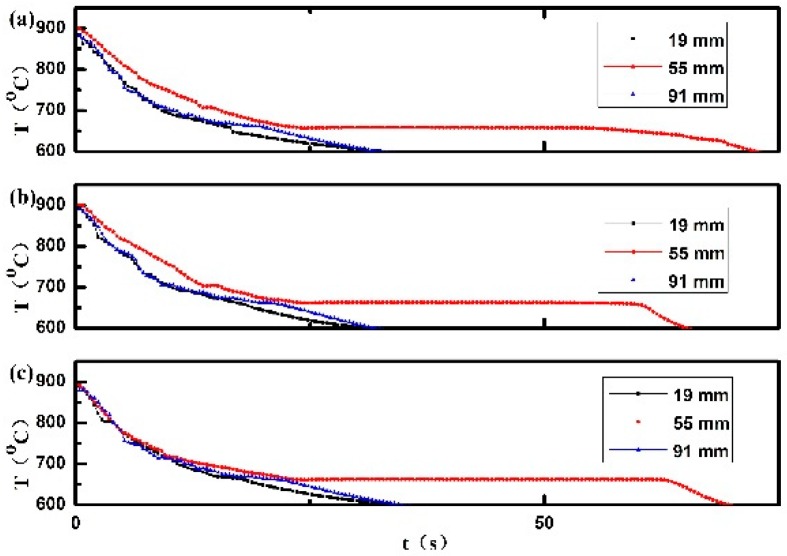
Cooling curves measured during solidification of pure aluminum: (**a**) without electropulsing; (**b**) under an alternating electropulsing with 480 A and 50 Hz and (**c**) under an alternating electropulsing with 900 A and 50 Hz.

For cooling behaviors of alloys under the condition of electromagnetically driven convection, it is noted that the melt flow makes a distinct reduction of temperature gradient and the turbulent melt flow correspond to the oscillations of the temperature signals [3,36]. Since the influence of the same electropulsing on the temperature field with mold preheated to different temperature was found to be qualitatively identical, it is decided to give the cooling curves obtained with mold at room temperature in the following discussion in order to determine the flow pattern. The cooling curves obtained from temperature measurements at three thermocouples placed along the longitudinal axis of ingots without and with electropulsing are given in Figure 6. Without electropulsing, an obvious axial temperature gradient exists in the aluminum melt from the beginning of directional solidification. However, the effect of electropulsing with 480 A on the axial thermal gradient can be neglected, as displayed in Figure 6b. It suggests that the obvious melt flow does not occur in this case. Therefore, the formation mechanism of FEG in pure aluminum should be different from the previous model [3,19,20,22,37]. When the electropulsing with 900 A is applied, a distinct reduction of the temperature gradient becomes visible implying a thermal homogenization of aluminum melt, and there is no distinct disturbance on cooling curves. It reveals the existence of laminar melt flow spanning the entire melt volume, which is essential for the formation of FEG in alloys [3,19,32,33].

### 3.3. Refinement Mechanism under Electropulsing

#### 3.3.1. Macrostructure in Sand Mold Embedding the Mesh Tube under Electropulsing

As indicated above, the generation of FEG is probably not related to the weak convection caused by Lorentz force in the presence of electropulsing with 480 A. Additionally, the role of Joule heating on grain refinement of pure aluminum is insignificant for the present electropulsing of ~53 A·cm^−2^ since effect of Joule heating on the solidification structure in Al-7Si alloy is almost negligible under electropulsing of ~120 A·cm^−2^ in Al-7Si alloy [3]. Thus, in the present study the observed formation of FEG is likely to be the consequence of electric current-associated free energy change Δ*G*_e_. When electric current passes through aluminum melt, the distribution of electric current is homogeneous. With reducing temperature, heterogeneous nuclei appear during the nucleation stage. Then the corresponding homogeneous distribution of electric current is modified because crystal nuclei have different electrical resistivity from that of the aluminum melt. Note that various distributions of electric current corresponds to the different *G*_e_, and thus Δ*G*_e_ is given as [28,29,38]:

(4) $$\Delta G_{e}=\frac{\mu }{8\pi }\iint \frac{j_{1}(r)j_{1}(r^{\prime })-j_{2}(r)j_{2}(r^{\prime })}{\left|r-r^{\prime }\right|}d^{3}rd^{3}r^{\prime },$$

where ***r*** and ***r*′** are two different positions in space, ***j*** is the current density and *μ* is the magnetic permeability. The subscript 1 and 2 represent the states before and after the formation of crystal nuclei. Equation (4) indicates that various distributions of electric current correspond to different Δ*G*_e_. Since both the occurrence of crystal nucleus and the different configuration of crystal nucleus affect the distribution of electric current, Δ*G*_e_ is derived by summing the volume and motion terms for electric current-associated free energy change:

(5) $$\Delta G_{e}=\Delta G_{\mathrm{eV}}+\Delta G_{\mathrm{em}}.$$

Assuming crystal nucleus of Al phase is a spherical form. If this nucleus occurs at the center of a spherical melt matrix under electropulsing, the general expression of the volume term can be given as [39]:

(6) $$\Delta G_{\mathrm{eV}}=k\frac{\sigma_{1}-\sigma_{2}}{2\sigma_{1}+\sigma_{2}}j^{2}V,$$

and

(7) $$k=\mu [\ln(\frac{R}{a})-\frac{65}{48}-\frac{5}{48}\frac{\sigma_{1}-\sigma_{2}}{2\sigma_{1}+\sigma_{2}}]R^{2},$$

where *σ*_1_ and *σ*_2_ are electric conductivities of the melt and crystal nuclei, respectively, *R* is the radius of the spherical melt-matrix, *k* is a positive geometry factor and *a* is the critical radius of crystal nucleus of Al phase, *V* is the volume of crystal nucleus. According to Equation (7), Δ*G*_eV_ will be negative due to *σ*_1_ < *σ*_2_. It means that the application of electropulsing reduces the change of system Gibbs free energy change and thus will help to promote heterogeneous nucleation in theory. However, effect of electropulsing on the nucleation rate is obviously insignificant in the nucleation stage of pure aluminum [20], i.e., Δ*G*_eV_ is much smaller than the change of Gibbs free energy between the liquid and solid phase.

If the spherical nucleus appears off-center, the crystal nucleus is driven from the initial position towards the center or surface of ingot by a force ***F*** arising from electropulsing. It can be expressed as [40]:

(8) $$F=-\frac{3}{2}\frac{\sigma_{1}-\sigma_{2}}{2\sigma_{1}+\sigma_{2}}\zeta j\times BV,$$

where ζ is the geometry factor and ***B*** is the magnetic intensity. The force ***F*** points from the outer surface to the center of ingot and in the perpendicular direction of electric current due to *σ*_1_ < *σ*_2_. Assuming crystal nucleus moves from one position *d* to the center of ingot, Δ*G*_e_ will tend to decrease. The corresponding decrease in value is equal to the work of the above force:

(9) $$\Delta G_{\mathrm{em}}=-\frac{3}{2}\frac{\sigma_{1}-\sigma_{2}}{2\sigma_{1}+\sigma_{2}}\mu_{0}\zeta j^{2}d^{2}V,$$

where *d* is the perpendicular distance from the position of crystal nucleus in space to the central axis of ingot. In our case, heterogeneous nucleation first occurs at the side wall and the interface of the electrodes and the melt. Since electropulsing is applied during the solidification process, the system free energy is related to the distribution of crystal nuclei during nucleation stage. As a result, crystal nuclei will move towards the central axis of ingot to minimize the Gibbs free energy of system. It is assumed that Δ*G*_em_ makes crystal nuclei appear at the side wall and the interface of the electrodes and the melt dissociate and drift in the melt, leading to the generation of FEG in ingot.

In order to check the validity of this assumption, it is of critical important that confirming where FEG originate from. Subsequently, a series of experiments with the mold embedded the mesh plate and tube of 06Cr25Ni20 stainless were designed. It is noted that the mesh plate and tube can separate crystal nuclei transfer [2,20,41], and suppress convection inside and outside the tube. Hence, the comparison of the solidification structure at the different regions is performed to clarify the role of Δ*G*_em_ on grain refinement in pure aluminum.

**Figure 7:**
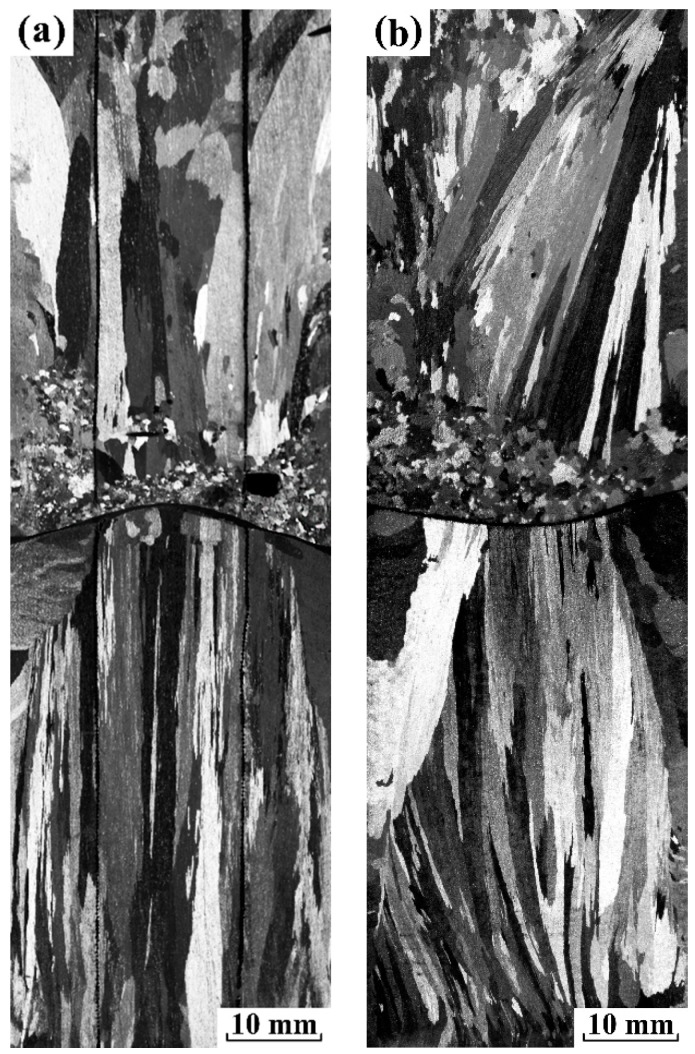
The solidification macrostructure along the longitudinal section of pure aluminum ingots under electropulsing with 480 A with a mold of 34 mm embedded the metal mesh: (**a**) a mesh plate located in the middle of mold and two mesh tubes embedded along axis and (**b**) a mesh plate placed in the middle of mold.

As shown in Figure 7, the experiment study on confirming the origin of FEG were designed under electropulsing with 480 A. For the first experiment, the applied sand mold consisted of a mesh plate located in the middle of mold and two mesh tubes embedded along axis. For another one, a mesh plate was only placed in the middle of mold. Moreover, both the solidification condition and the applied electropulsing parameters were the same as those of experiment 3 (a). Figure 7 illustrates the corresponding longitudinal sections of the solidification structure of pure aluminum ingots. It is observed that the fine equiaxed zone existed ahead of the vertical downward columnar grains above the mesh plate regardless of inside or outside the tube, as shown in Figure 7a. In contrast, the dominant columnar grains with vertical upward direction appeared below the mesh plate. In Figure 7b, the macrostructure above and below the mesh plate was similar to the corresponding one in Figure 7a. It is noted that current effects could be divided into similar halves along the mesh plate in our case. However, the direction of grain growth above and below the mesh plate reversed during the solidification process. From the macrostructural comparison between the vertical upward and downward directional solidification of Al subjected to electropulsing, there was no doubt that FEG above the mesh plate originated from the dissociation of heterogeneous nuclei on the upper interface of the electrode and the melt. These results were very similar to those presented by Li et al. [19] in which the nuclei detachment is ascribed to the visible shock wave driven by the periodic Lorentz force. As has been reported before, the mesh tube has an effect on restricting the melt flow inside and outside the tube [20]. If the weak forced flow dominates the formation of FEG in our case, the total amount of FEG inside and outside the tube should obviously decrease, which is in contradiction with the macrostructural observation. It suggests that the weak forced flow had no effect on grain refinement, and thus the dissociation of crystal nuclei on the upper interface was solely attributed to Δ*G*_em_.

**Figure 8:**
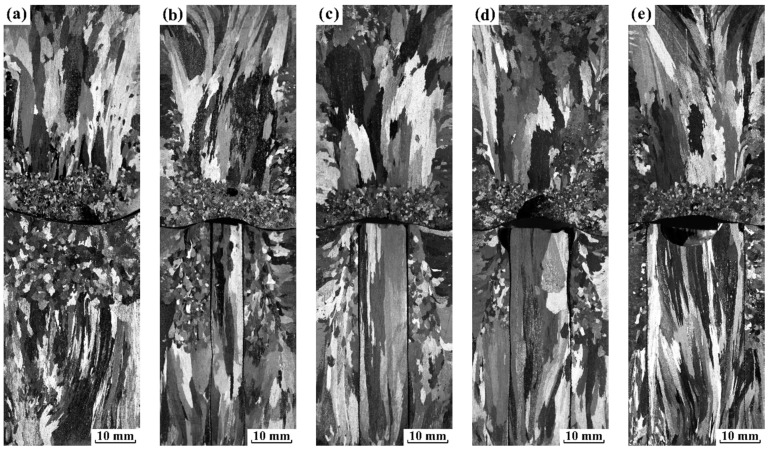
The solidification macrostructure along the longitudinal section of pure aluminum ingots under electropulsing with 900 A with mold of 34 mm embedded a mesh plate located in the middle of mold and a mesh tube of different diameter embedded along axis: (**a**) without the tube; (**b**) the tube of 8 mm; (**c**) the tube of 12 mm; (**d**) the tube of 16 mm and (**e**) the tube of 24 mm.

As current intensity increased to 900 A, the origin of FEG refers to a more complex picture including Δ*G*_em_ on the solidification process and the forced flow. Thus, five experiments were designed to explain the role of Δ*G*_em_ and the forced flow on grain refinement in pure aluminum. For each experiment, sand mold consists of a mesh plate located in the middle of mold and a mesh tube with different diameters embedded along axis. The diameter of mesh tube was 0, 8, 12, 16 and 24 mm, respectively. Moreover, both the solidification condition and the applied electropulsing parameters were the same as those of experiment 4 (a). The longitudinal as-cast macrostructure of pure aluminum ingots using different molds is displayed in Figure 8. Without the mesh tube, a significant fine equiaxed zone appeared in the front of vertical downward columnar grains above the mesh plate and vertical upward columnar grains below the mesh plate. However, the equiaxed grain above the mesh plate was remarkably finer compared with those below the mesh plate. When a tube with 8 mm in diameter was embedded, the macrostructure changed little above the mesh plate. Below the mesh plate, columnar grains expanded over the entire height of mesh tube inside it, and a fine equiaxed zone occurs outside the tube. An increase in the diameter of mesh tube to 12 mm (Figure 8c), 16 mm (Figure 8d) or 24 mm (Figure 8e) decreased the amount of FEG outside the tube, but the coarse columnar grains still dominated the macrostructure inside the tube. The observed change of fine equiaxed zone below the mesh plate in the present study was quite similar to those presented by previous work [20]. It implies that the forced convection plays an important role in the generation of FEG. However, it could not be determined to whether Δ*G*_em_ will give rise to the dissociation of heterogeneous nuclei on the side wall or not.

**Figure 9:**
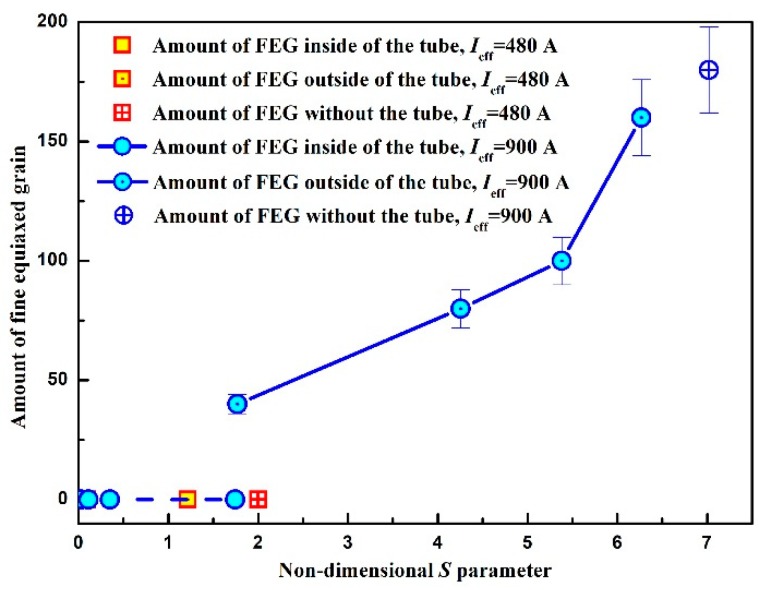
Relationship between *N* in different regions of mold embedded stainless mesh below the mesh plate and non-dimensional *S* parameter.

As discussed above, the formation of FEG should attribute to competition and cooperation between Δ*G*_em_ and the forced melt flow. To clarify this speculation, the amount of FEG in different regions of mold embedded stainless mesh below the mesh plate *N* was measured with respect to the different melt flow. Figure 9 represents the variation in *N* as a function of non-dimensional *S* parameter, which was calculated based on Equation (3). Here, the value *ν* = 1.25 mPa·s [42] and *ρ* = 2.35 g·cm^−3^ [43] were employed. It can be seen that *N* gradually increased with the *S* parameter outside the tube under the same electropulsing of 99.2 A·cm^−2^, and there was no the formation of FEG inside the tube in the present experiments. When the calculated *S* parameter was approximately 1.77 FEG occurred outside the tube. In contrast, there was no the formation of FEG beyond this value under electropulsing of 52.9 A·cm^−2^. Since the driving force Δ*G*_em_ was proportional to current density, Δ*G*_em_ under electropulsing of 52.9 A·cm^−2^ was probably insufficient to drive the dissociation of heterogeneous crystal nuclei at the lateral wall (Figure 7b). Meanwhile, the present melt flow had little effect on the formation of FEG. This speculation explains the experimental data under electropulsing of 52.9 A·cm^−2^ very well. It is noted that even if the intensity of forced convection was much smaller than in the preceding case, FEG still appeared near the mold wall under electropulsing of 99.2 A·cm^−2^ (Figure 8e). This indicates that the higher driving force Δ*G*_em_ gave rise to the dissociation of crystal nuclei on the side wall, leading to the generation of FEG. For a further increase in the intensity of the melt flow (Figure 8a–d) the additional FEG arising from the dissociation of crystal nuclei on the side wall was induced by the stronger convection.

**Figure 10:**
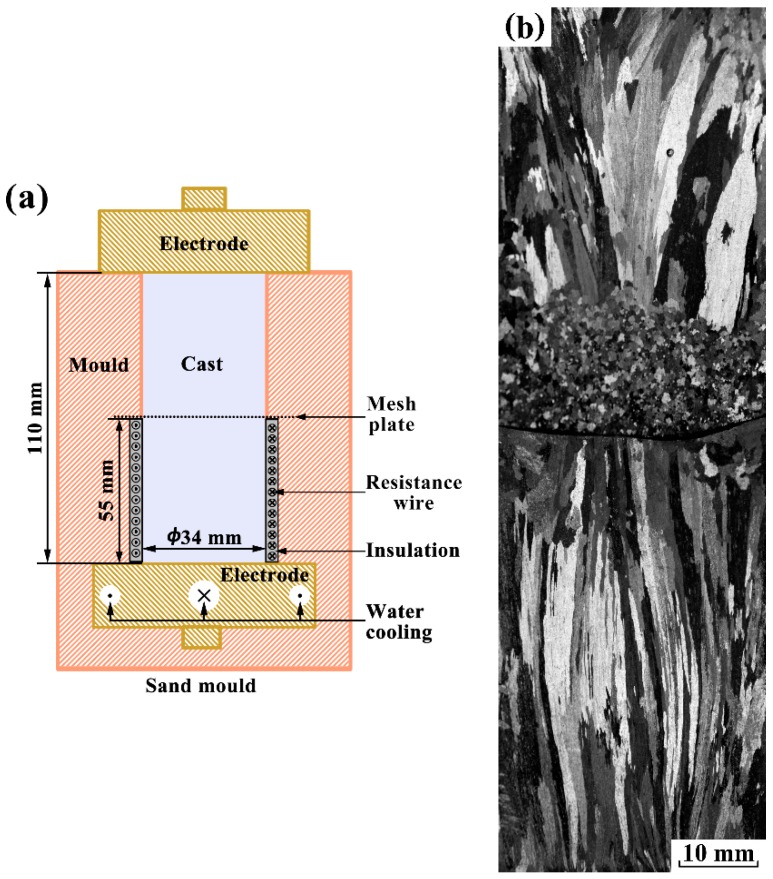
(**a**) Schematic sketch of the improved mold and (**b**) longitudinal sections of the solidification structure in pure aluminum ingots under electropulsing with 900 A with the improved mold.

As described in the introduction, the strong forced flow can induce dendrite fragmentation, leading to the formation of FEG [20,21]. However, the present analysis cannot identify whether dendrite fragmentation is distinctly induced by the application of electropulsing with 900 A. In order to explain it, a casting experiment with an improved mold was designed. As shown in Figure 10a, the mold was separated into two parts by a horizontally placed mesh: the sand mold with 34 mm in diameter and 55 mm in height above the mesh, the ceramic mold with 34 mm in internal diameter, 55 mm in height and a 3 mm in thickness below the mesh. The outer surface of the ceramic mold was twined by heated Cr20Ni80 resistance wire to prevent the radial heat flux through the mold. Here, the temperature of the side wall was preheated to 933 K, both the solidification condition and the applied electropulsing parameters were same as those of experiment 4 (a). The longitudinal as-cast macrostructure of pure aluminum ingots using an improved mold is displayed in Figure 10b. As a result, a significant fine equiaxed zone appears in the front of vertical downward columnar grains above the mesh plate. However, the coarse columnar grains take up the entire ceramic mold. Below the mesh plate, crystal nuclei appeared at the bottom electrode and developed into columnar structure during the solidification process. If FEG generates in this case, its dominant origin should be dendrite fragmentation caused by the melt flow. From macrostructural observation, it could confirm that the application of electropulsing with 900 A could not lead to the significant fragmentation of columnar grains.

#### 3.3.2. Formation of FEG

In view of the above investigation, the following conclusions were demonstrated.

(1) The amount of FEG remains almost unchanged as the preheated temperature of mold increased. It confirmed that the dissociation of heterogeneous nuclei formed on the side wall did not occur under electropulsing with 480 A (Figure 3).
(2) A partial FEG result from the dissociation of heterogeneous nuclei formed on the lateral wall when electropulsing with 900 A was applied (Figure 4).
(3) The obvious melt flow did not occur under electropulsing with 480 A. However, the application of electropulsing with 900 A could lead to a laminar melt flow spanning the entire melt volume (Figure 6).
(4) Since the forced melt flow was not taken into account under electropulsing with 480 A, the formation of FEG was attributed to the dissociation of heterogeneous nuclei on the upper interface driven by Δ*G*_em_.
(5) The origin of FEG consisted of the dissociation of heterogeneous nuclei on the side wall and the upper interface due to Δ*G*_em_ and the forced melt flow under electropulsing with 900 A.

**Figure 11:**
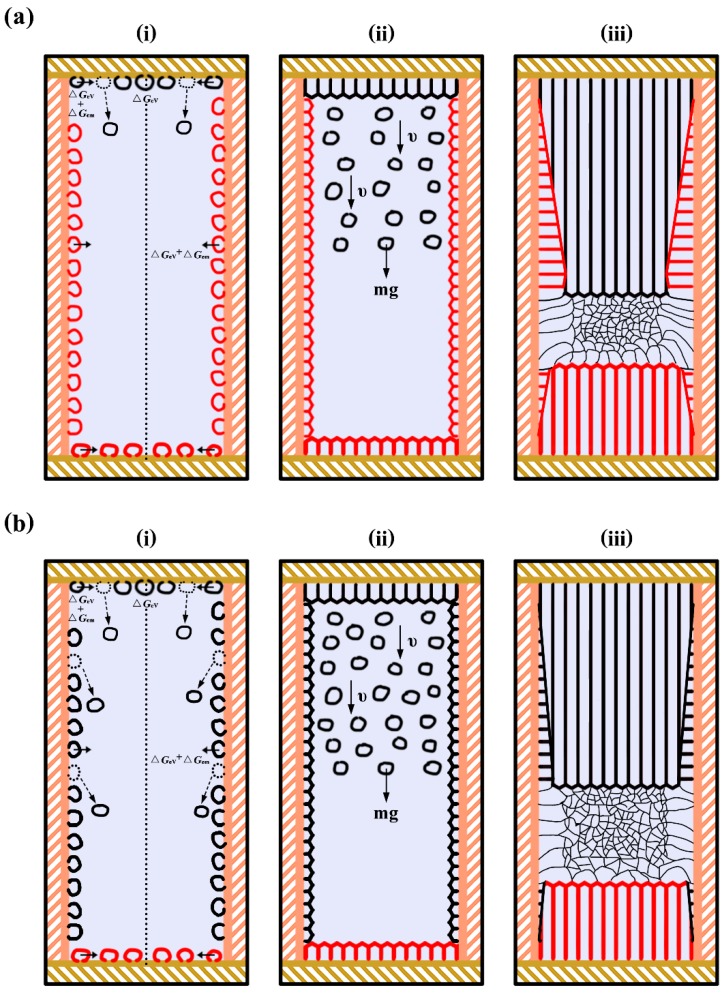
Schematic illustration of the formation process of FEG observed in the present study. The solidification proceeds from (**i**) to (**iii**). (**a**) under electropulsing with 480 A and (**b**) under electropulsing with 900 A.

From these findings, the following scenario was considered for the formation process of FEG during solidification of pure aluminum with the application of present electropulsing, as schematically illustrated in Figure 11. Under electropulsing with 480 A, heterogeneous nuclei firstly appeared on the side wall and the interface of the electrodes and the melt. The applied electropulsing made heterogeneous nuclei fall off from the upper interface until the solidification shell forms due to Δ*G*_em_, as shown in Figure 11ai. Then, the dissociated crystal nuclei moved towards the bottom of the mold due to its gravity and the grains in the solidification shell, which could grow parallel and opposite to the heat flow direction, would develop into a columnar structure (Figure 11aii). Subsequent, columnar-to-equiaxed transition occurred, if the number and size of the equiaxed grains ahead of the upward and downward columnar grain front accumulated to their limit, i.e., the columnar growth was blocked and an equiaxed zone formed, as shown in Figure 11aiii. Similar results have been reported in earlier studies [44,45]. The formation process of FEG under electropulsing with 900 A is schematically illustrated in Figure 11bi–iii. Compared with the proposed process under electropulsing with 480 A, the main distinction was that FEG grew from the dissociated crystal nuclei, which resulted from the lateral wall and the upper interface of the electrode and the melt due to Δ*G*_em_ and the forced convection under electropulsing with 900 A, as shown in Figure 11bi.

As indicated above, the driving force Δ*G*_em_ was one of the important factors of inducing the dissociation of crystal nuclei in the present study. However, the dissociation of crystal nuclei caused by Δ*G*_em_ on the upper interface was easier than that on the lateral wall, probably due to the difference in the direction of *F* imposed to the crystal nuclei attachment surface. As has been reported before, the direction of *F* is axial symmetrical and from the center of ingot to the lateral surface when the electrical conductivity of inclusions is less than that of the matrix [28]. Since the electrical conductivity of crystal nuclei (~10^5^ S·cm^−1^ at 873 K [46]) was more than that (~4 × 10^4^ S·cm^−1^ at 973 K [46]) of the molten aluminum, *F* with the reversed direction was produced in our case. In other words, the direction of *F* pointed towards the central axis and was parallel to the upper interface of the electrode and the melt when the force was imposed to crystal nuclei on the substrate of the upper interface. In contrast, the direction was perpendicular to the surface of mold for the force applied to crystal nuclei on the substrate of lateral wall. From the macrostructural observation, it confirmed that the required shear stress on the upper interface of the electrode and crystal nuclei for the dissociation of crystal nuclei was less compared to the normal stress on the contact surface of the lateral wall and crystal nuclei.

Under electropulsing with 900 A, the proposed process for the dissociation of crystal nuclei on the side wall was the result of Δ*G*_em_ in cooperation with the forced convection, which was distinctly different from the contribution of magnetic pressure pulses presented by Liao et al. [3]. It is noted that the non-dimensional *S* parameter utilized to characterize the intensity of forced convection was proportional to (*jR*^2^)^2^, and the maximum value of Δ*G*_em_ varied as (*jR*)^2^. Based on these different functional dependencies, we could conclude the following: (i) there was a threshold value of current density, below which the forced melt flow would make the dissociation of crystal nuclei and thus led to grain refinement before Δ*G*_em_ had any effect on crystal nuclei on the upper surface. The effect of Δ*G*_em_ on grain refinement will have a priority if current density exceeds the threshold value. This analysis was strongly supported by the macrostructural observation shown in Figure 7; (ii) another higher threshold value of current density existed. Δ*G*_em_ firstly dominated the dissociation of crystal nuclei on the upper interface and the side wall before the forced convection played a role in forming the refined grains if current density exceeded this value; (iii) if current density and the size of the mold were large enough, both Δ*G*_em_ and the forced convection played important roles in the dissociation of crystal nuclei on the side wall for grain refinement, which were in agreement with the analysis for the solidification structure illustrated in Figure 8a–d. Therefore, the model of the combination of Δ*G*_em_ and the forced melt flow for grain refinement explained the macrostructural difference below the mesh plate shown in Figure 7 and Figure 8, and strongly supported the validity of the proposed scenario in Figure 11.

**Figure 12:**
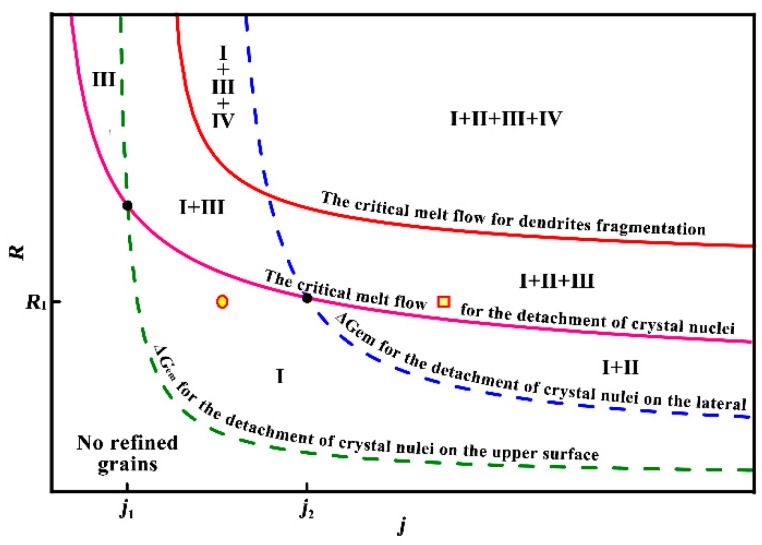
Schematic summary of the origin of FEG in pure aluminum under electropulsing. For the origin of FEG, I, III and IV represent the dissociation of crystal nuclei on the upper interface of the electrode and the melt due to Δ*G*_em_, the dissociation of crystal nuclei on the side wall due to Δ*G*_em_, the dissociation of crystal nuclei due to melt flow and dendrite fragmentation, respectively.

It is noted that the scenario of the FEG formation process proposed here essentially relied on Δ*G*_em_ and the forced melt flow. As described in the introduction, previous studies [5,20,21] demonstrated that dendrite fragmentation induced by the strong forced convection was one of the main origins of FEG. Therefore, the mechanism of grain refinement considered comprehensively factors in terms of the dissociation of crystal nuclei due to Δ*G*_em_, and the dissociation of crystal nuclei and dendrite fragmentation due to the forced melt flow. Figure 12 shows the schematic summary of the origin of FEG in pure aluminum under electropulsing. *j* and *R* were the main variables that determined the origin of FEG in a normal casting. The dash curves represent a constancy of the origin of FEG induced by Δ*G*_em_ (no effect, the dissociation of crystal nuclei on the upper interface, and on the side wall). Additionally, the various values (solid curves running from upper left to lower right) represent a constancy of the origin of FEG induced by the forced melt flow (no effect, the dissociation of crystal nuclei and dendrites fragmentation). The different regions segmented by curves correspond to the different origins of FEG. Thus, the role of Δ*G*_em_ and the forced melt flow on the origin of FEG in pure aluminum under electropulsing could be determined. The circle and square symbol were superimposed on the graph to indicate the origin of FEG under electropulsing of 52.9 and 99.2 A·cm^−2^, respectively.

#### 3.3.3. The Solidification Macrostructure under Different Conditions

According to the analysis above, the formation of FEG observed in these simples were attributed to Δ*G*_em_ and the forced convection driven by Lorentz force. Furthermore, which current effects play the dominant roles for grain refinement of pure aluminum depends on the current density and the size of the mold. To check the validity of the proposed process, the concluded results that have not been demonstrated should be verified experimentally by controlling Δ*G*_em_ and the forced melt flow.

**Figure 13:**
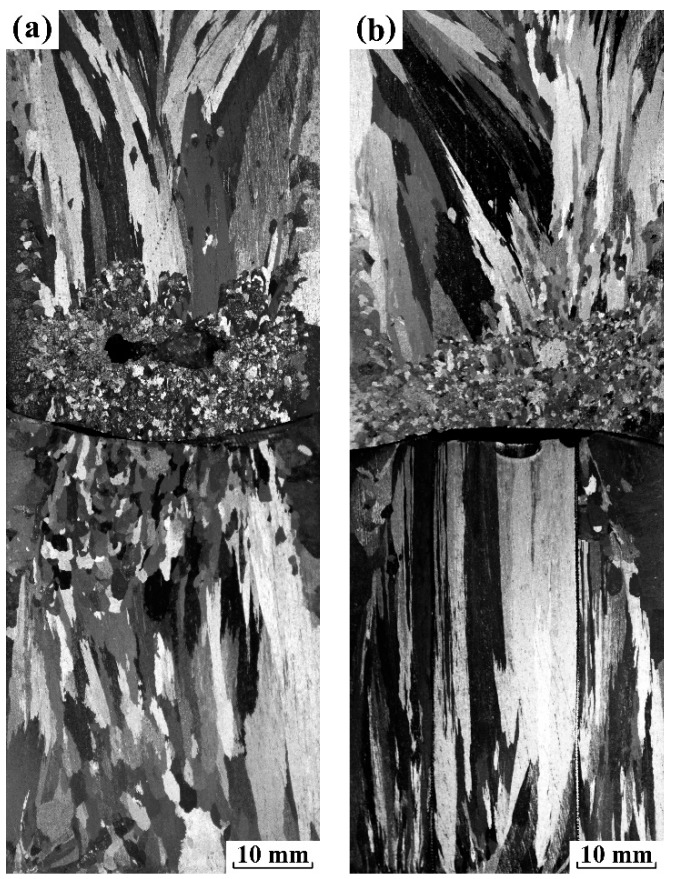
The longitudinal solidification macrostructure in pure aluminum ingots under electropulsing of 700 A with mold of 40 mm in diameter embedded the metal mesh: (**a**) a mesh plate located in the middle of mold and (**b**) a mesh plate embedded along the cross section and a mesh tube embedded along axis.

As indicated by Figure 12, the forced convection might give rise to the dissociation of crystal nuclei besides the dissociation of crystal nuclei on the upper interface due to Δ*G*_em_ under electropulsing of 52.9 A·cm^−2^ as increasing the radius of ingots, which was proved by the two following casting experiments. Here, the applied current intensity was 700 A and the size of the sand mold was changed compared with that in the casting experiments under electropulsing (Figure 1). For the first experiment, the cylindrical sand mold with 40 mm in diameter was selected and a mesh plate was located in the middle of mold in this case. For another one, the sand mold consisted of a mesh plate embedded along the cross section and a mesh tube with 20 mm in diameter embedded along axis. The corresponding solidification macrostructure is shown in Figure 13. Obviously, a significant fine equiaxed zone appeared ahead of the downward columnar front above the mesh plate and upward columnar front below the mesh plate, respectively. However, the equiaxed grains above the mesh plate were obviously finer compared with those below the mesh plate. When a mesh tube was embedded, coarse columnar grains took up the entire mesh tube inside and outside it below the mesh plate. Based on Equation (3), the non-dimensional *S* parameter without the mesh tube was calculated to be 4.25, which exceeded the required value (<4.26) for the dissociation of crystal nuclei on the side wall and was below the critical value (>7.02) for dendrite fragmentation due to melt flow. It suggests that FEG below the mesh plate resulted from the dissociation of crystal nuclei on the side wall due to the forced convection. The *S* parameter was also calculated to be 0.27 and 2.39 inside and outside the tube, respectively. These values were below the critical value for the dissociation of crystal nuclei on the side wall due to forced convection. From the macrostructural observation (Figure 13), it confirmed that Δ*G*_em_ had no effect on the formation of FEG below the mesh plate, and the forced convection solely led to the formation of FEG originated from the dissociation of crystal nuclei on the side wall in this case, which was identical with the theoretical analysis. Therefore, the role of Δ*G*_em_ and the forced convection on grain refinement in pure aluminum mainly depended on the size of the mold and current density.

## 4. Conclusions

In the present study, the effect of an alternating electropulsing on grain refinement in pure aluminum was investigated by the designed casting experiments. Although the application of electropulsing can induce various kinds of current effects including Joule heating, Δ*G*_em_ and the forced convection are particularly worth considering for grain refinement. Correspondingly, the origin of FEG is different from the conventional assumption for the dissociation of crystal nuclei on the side wall and dendrite fragmentation induced by the forced convection. The important findings could be drawn as follows.

(1) During solidification in the present casting, crystal nuclei firstly generated at the upper interface of the electrodes and the melt and the side wall. The driven force Δ*G*_em_ was the first to have an effect on grain refinement due to the dissociation of crystal nuclei on the upper interface if *j* > *j*_1_, which Δ*G*_em_ or the forced convection had a priority to the dissociation of crystal nuclei at the side wall depended on *j*_2_ in Figure 12.
(2) There was also a critical value for the forced melt flow, above which the dissociation of crystal nuclei at the upper interface of the electrodes and the melt and the side wall was induced and developed into FEG. With the increase of the forced melt flow, dendrites fragmentation during crystal growth occurred and became a significant factor for grain refinement.
(3) Current density and the size of mold were two main parameters for grain refinement under electropulsing, which determined Δ*G*_em_ and the forced convection, affected the origin of FEG.
